# Association between axial length and HDL in children: a hospital-based cross-sectional study

**DOI:** 10.1186/s12886-023-02902-8

**Published:** 2023-04-18

**Authors:** Qingqing Zheng, Chaoyang Hong, Yaling Peng, Ting He, Yuan Lai, Lingtong Tan, Ting Shen

**Affiliations:** 1 Center for Rehabilitation Medicine, Department of Ophthalmology, Zhejiang Provincial People’s Hospital, Affiliated People’s Hospital, Hangzhou Medical College, Hangzhou, Zhejiang China; 2 School of Ophthalmology and Optometry, Wenzhou Medical University, Wenzhou, Zhejiang China; 3 Eye Center, the Second Affiliated Hospital, School of Medicine, Zhejiang University, 88 Jiefang Road, Hangzhou, 310009 Zhejiang China

**Keywords:** Axial length, High-density lipoprotein, Children, Myopia, Ocular

## Abstract

**Background:**

To analyze the relationship between axial length and levels of high-density lipoprotein (HDL) cholesterol in children.

**Methods:**

A retrospective, hospital-based cross-sectional research with 69 right eyes from 69 children who underwent health examination by Zhejiang Provincial People’s Hospital was carried out. The participants were split into three groups: Group A (axial length < = 23 mm), Group B (axial length 23–24 mm), and Group C (axial length > 24 mm). Demographic epidemiological information, blood biochemical parameters and ophthalmic characteristics including refractive status and ocular geometric parameters were obtained and analyzed.

**Results:**

69 right eyes from 69 patients (25 males and 44 females) with a median age of 10.00 years old (IQR: 8.00–11.00 years) were included in the study. Within Group A, there were a total of 17 individuals; Group B consisted of 22 individuals; Group C included 30 individuals. The mean axial length of three groups was 22.148(0.360), 23.503(0.342) and 24.770(0.556) mm, respectively (*p* < 0.0001). The mean HDL levels were significantly different in three groups are 1.824(0.307), 1.485(0.253) and 1.507 (0.265) mmol/L, respectively. By applying a Pearson Coefficient, we evaluated the association between axial length and HDL and discovered that there was a statistically significant (*p* = 0.00025) and adverse (R = -0.43) association between axial length and HDL.

**Conclusions:**

We concluded from our study that there was a significantly inverse relationship between axial length and the levels of HDL in children.

## Background

Myopia is the most prevalent visual disorder throughout the globe. In general, visual acuity can be maintained at a normal level by wearing eye glasses, contact lens or by refractive surgical procedures. In the past 70 years, myopia has been steadily becoming worse [1]. By 2050, it is predicted that myopia would affect half of the world’s population [2]. The situation has grown extremely complicated especially for East Asia [3–5]. It has caused serious social and economic burdens [6–9]. Axial length (AL) elongation is a crucial endophenotype of myopia because it plays a significant role in the development and progression of myopia [10].

Many factors can influence the development of the axial length. Axial elongation is mostly influenced by age. In the first 2 to 3 years after birth, the human eyeball experiences fast postnatal growth. During this time, the axial length typically stretches from around 16 to 17 mm to 22.5 to 23 mm [11, 12]. Following this, the expansion of axial length slows down and it eventually achieves its maximum size, with a further increment of 1 mm occurring until around 13 years of age [13, 14]. Austin Bach found that the mean axial length was 19.7643 mm for 3–6 months old baby, and growth in axial length were greatest in the first 10 months of life and continued beyond that, but at a slower pace, to 22.4156 mm at age 6–7 years old for American children [15]. Xiangui He reported that at 4 years old, axial length was 22.50 mm and 21.88 mm for boys and girls respectively, and it grew at a steady speed to 25.23 mm and 24.99 at the age of 18 years old for Chinese children [16]. Other factors associated with axial includes sex, height, education, gene, environmental factors and so on [17].

Some serum metabolomics, including phospholipid, diacylglycerol, amino acid, vitamin metabolism, γ-glutamyltyrosine and 12-oxo-20-trihydroxy-leukotriene B4 have been proved to be related to myopia [18]. A positive association between myopia and melatonin have been demonstrated [19]. A negative relation between 25(OH)D concentration and risk of myopia has also been found [20]. High-density lipoprotein (HDL), one of the five main subgroups of lipoproteins, however, has not been investigated whether there exists some relation with myopia. By acting as a scavenger for extra cholesterols and preventing their buildup, it is usually known as a healthy cholesterol. In other words, HDL can carry these cholesterols from the arterial tissues to the liver for biliary excretion and breakdown. Additionally, it prevents thrombosis, endothelial inflammation, and low-density lipoprotein (LDL) oxidation [21, 22].

Regardless of this, to the best of our understanding, there isn’t enough information about the relationship between axial length and high-density lipoprotein (HDL) cholesterol. Herein, we investigated the relationship between axial length and HDL in children in a Chinese hospital.

## Methods

### Study population

A cross-sectional study was carried out between January 2020 and January 2021 in the Department of Ophthalmology, Zhejiang Provincial People’s Hospital, Hangzhou, China. The principles of the Declaration of Helsinki were followed at every step of the process. Prior to the trial, all participants provided written informed consent. Informed consent to participate in the study was obtained from participants and their parent or legal guardian. Our study was approved by the Ethics Committee of Zhejiang Provincial People’s Hospital.

From January 2020 and January 2021, all children aged 7–13 who underwent a health examination in our department were asked to participate. The following criteria were used to exclude people: children who weren’t able to provide their consent for the study; children who were now suffering from eye infection conditions including conjunctivitis, keratitis, uveitis; children who were diagnosed with any serious ophthalmic disorders, such as congenital cataracts, glaucoma, strabismus, anisometropia or amblyopia; children who were diagnosed with short stature or precocious puberty; children who experienced psychological discomfort when evaluating a physical parameter.

### Ophthalmic examination

All of the participants received a comprehensive ocular examination, including slit lamp and dilated fundus exams. After administering full cycloplegia to both eyes, the spherical equivalent refraction (SER) was determined with the assistance of an autorefractometer (KR-800, Topcon Corporation, Tokyo, Japan). With the use of Snellen VA charts, best-corrected visual acuity (BCVA) was performed. BCVA was then transformed to the logarithm of the minimal angle of resolution (logMAR). Corneal topography (Orbscan II, Bausch & Lomb, Rochester, NY) was applied to acquire the corneal power (steepest keratometry SK, flattest keratometry FK, and mean keratometry Km). Axial length was obtained using the Zeiss IOLmaster 500 (Carl Zeiss Meditec AG, Jena, Germany). After at least 8 h of fasting, blood samples were taken, and cholesterol levels of HDL were measured using HLC-729LPII (Tosoh Corporation) based on AEX-HPLC method. Other fundamental factors including age, sex, height, weight, BMI, thyroid function, sex hormone and main biochemical parameters were also measured.

### Statistical analysis

R software (version 4.2.1) was used to conduct the statistical analysis. Shapiro-Wilk tests were run on each variable and sample to see if they were regularly distributed. Continuous data are presented as means +- standard deviation (SD) for variables with a regularly distributed distribution and as medians and interquartile range (25th to 75th percentile) for variables with a non-normal distribution. When comparing two groups, a two-tailed unpaired t-test was used for the statistical analysis, and when comparing three groups or more, an ANOVA was used for HDL. The association between the concentration of HDL and the axial length was examined using the Pearson correlation test.

## Results

### General characteristics

Our study included 69 right eyes from 69 patients (25 males and 44 females) having a median age of 10.00 years old (IQR: 8.00–11.00 years). The median BCVA was 1.0 (IQR: 1.0-1.2). These patients were divided into 3 groups according to the axial length: axial length < = 23 mm (Group A), 23 mm < axial length < = 24 mm (Group B) and axial length > 24 mm (Group C). Finally, within Group A, there were a total of 17 individuals; Group B consisted of 22 individuals; Group C included 30 individuals. The mean axial length in three groups was 22.148(SD = 0.360), 23.503(SD = 0.342) and 24.770(SD = 0.556) mm, respectively (p < 0.0001). Regarding to anterior chamber depth, it was 3.320(3.050, 3.470), 3.390(3.335, 3.495) and 3.630(3.512, 3.790) mm, respectively (p = 0.0001). HDL levels were 1.824 (0.307), 1.485 (0.253) and 1.507 (0.265) mmol/L, respectively. It was found that the HDL differed significantly among three groups(*p* = 0.0003). Other factors were also included and analyzed in our study. Tables 1 and 2 present a summary of the distribution of the general features. No significant differences in BCVA or sex were detected among groups (*p* > 0.05).

**Table 1:** General characteristics of all participants

Characteristics		Overall	Group A	Group B	Group C	p
n		69	17	22	30	
Gender, n (%)						0.2836
Male		25 (36.23)	5 (29.41)	6 (27.27)	14 (46.67)	
Female		44 (63.77)	12 (70.59)	16 (72.73)	16 (53.33)	
Age (years) (median [IQR])		10.000 [8.000, 11.000]	7.000 [5.000, 9.000]	9.000 [9.000, 10.000]	11.000 [9.250, 11.750]	< 0.0001
Diopter (D) (median [IQR])		-0.250 [-1.500, 0.250]	0.250 [0.000, 1.000]	0.000 [-0.875, 0.500]	-1.375 [-2.688, -0.250]	< 0.0001
BCVA (median [IQR])		1.000 [1.000, 1.200]	1.000 [1.000, 1.000]	1.000 [1.000, 1.200]	1.000 [1.000, 1.200]	0.1196
Axial Length (mm) (mean (SD))		23.720 (1.148)	22.148 (0.360)	23.503 (0.342)	24.770 (0.556)	< 0.0001
ACD (mm) (median [IQR])		3.480 [3.330, 3.650]	3.320 [3.050, 3.470]	3.390 [3.335, 3.495]	3.630 [3.512, 3.790]	0.0001
Bone Age (years) (median [IQR])		10.700 [9.600, 12.300]	6.100 [5.700, 10.200]	10.700 [10.600, 11.975]	11.750 [10.050, 12.700]	< 0.0001
BMI (kg/m^2^) (median [IQR])		17.830 [16.050, 19.200]	16.050 [15.300, 16.350]	18.155 [17.830, 22.825]	17.885 [16.093, 20.348]	0.0001
Axial Length(three groups) (%)						< 0.0001
<=23 mm		17 (24.64)	17 (100.00)	0 (0.00)	0 (0.00)	
> 24 mm		30 (43.48)	0 (0.00)	0 (0.00)	30 (100.00)	
23-24 mm		22 (31.88)	0 (0.00)	22 (100.00)	0 (0.00)	

BCVA: best-corrected visual acuity; ACD: anterior chamber depth.

**Table 2:** Main biochemical parameters of all participants

Characteristics	Overall	Group A	Group B	Group C	p
HDL (mmol/L)(mean (SD))	1.578 (0.303)	1.824 (0.307)	1.485 (0.253)	1.507 (0.265)	0.0003
LDL (mmol/L)(mean (SD))	2.158 (0.481)	2.207 (0.373)	2.051 (0.543)	2.209 (0.489)	0.4545
ICF.1 (ng/ml)(median [IQR])	435.000 [280.000, 551.000]	251.000 [161.000, 450.000]	507.000 [250.750, 584.000]	436.500 [370.000, 542.500]	0.0324
25-Hydroxyvitamin D3 (ng/mL)(mean (SD))	21.780 (7.086)	22.335 (7.682)	20.720 (6.861)	22.242 (7.059)	0.7022
TT3 (nmol/L)(mean (SD))	1.315 (0.145)	1.285 (0.138)	1.304 (0.144)	1.340 (0.151)	0.4223
TT4 (nmol/L)(mean (SD))	86.230 (10.156)	83.935 (8.599)	86.223 (5.551)	87.536 (13.188)	0.5125
TSH (mU/L)(median [IQR])	2.130 [1.540, 3.230]	2.130 [1.310, 2.450]	1.915 [1.570, 2.850]	2.340 [1.500, 3.458]	0.1806
TP (g/L)(median [IQR])	73.800 [68.200, 78.400]	72.800 [70.500, 75.300]	73.900 [67.050, 78.600]	75.700 [68.250, 78.250]	0.9713
AKP (U/L)(median [IQR])	282.000 [257.000, 334.000]	282.000 [274.000, 345.000]	254.000 [235.000, 318.000]	283.500 [262.500, 334.000]	0.2218
TCHO (mmol/L)(median [IQR])	3.880 [3.670, 4.530]	3.880 [3.450, 4.780]	3.790 [3.680, 4.520]	3.880 [3.655, 4.530]	0.74
TG (mmol/L)(median [IQR])	0.780 [0.690, 1.040]	0.860 [0.760, 1.400]	0.730 [0.690, 0.980]	0.755 [0.680, 0.857]	0.252
HCY (umol/L)(median [IQR])	9.400 [8.900, 10.200]	9.700 [8.300, 10.300]	9.500 [9.175, 10.200]	9.400 [8.750, 9.650]	0.5213
LH (miU/mL)(median [IQR])	0.950 [0.230, 2.040]	0.670 [0.080, 2.230]	1.160 [0.500, 1.588]	0.730 [0.360, 3.138]	0.7116
E2 (pg /mL)(median [IQR])	8.200 [8.000, 12.090]	8.700 [8.000, 11.560]	7.800 [7.000, 8.200]	8.900 [8.000, 20.625]	0.0092
P (ng /mL)(median [IQR])	0.100 [0.040, 0.140]	0.110 [0.030, 0.120]	0.050 [0.030, 0.110]	0.110 [0.050, 0.140]	0.12
T (ng /mL)(median [IQR])	0.180 [0.120, 0.430]	0.230 [0.120, 1.200]	0.125 [0.120, 0.262]	0.270 [0.130, 0.430]	0.0752

IGF-1: Insulin-like growth factor-1; TT3: total triiodothyronine; TT4: total thyroxine; TSH: thyroid-stimulating hormone; TP: total protein; AKP: alkaline phosphatase; TCHO: total cholesterol; TG: triglyceride; HCY: homocysteine; LH: luteinizing hormone; E2: estradiol; P: progesterone; T: testosterone

### Relationship between axial length and HDL

**Fig. 1:**
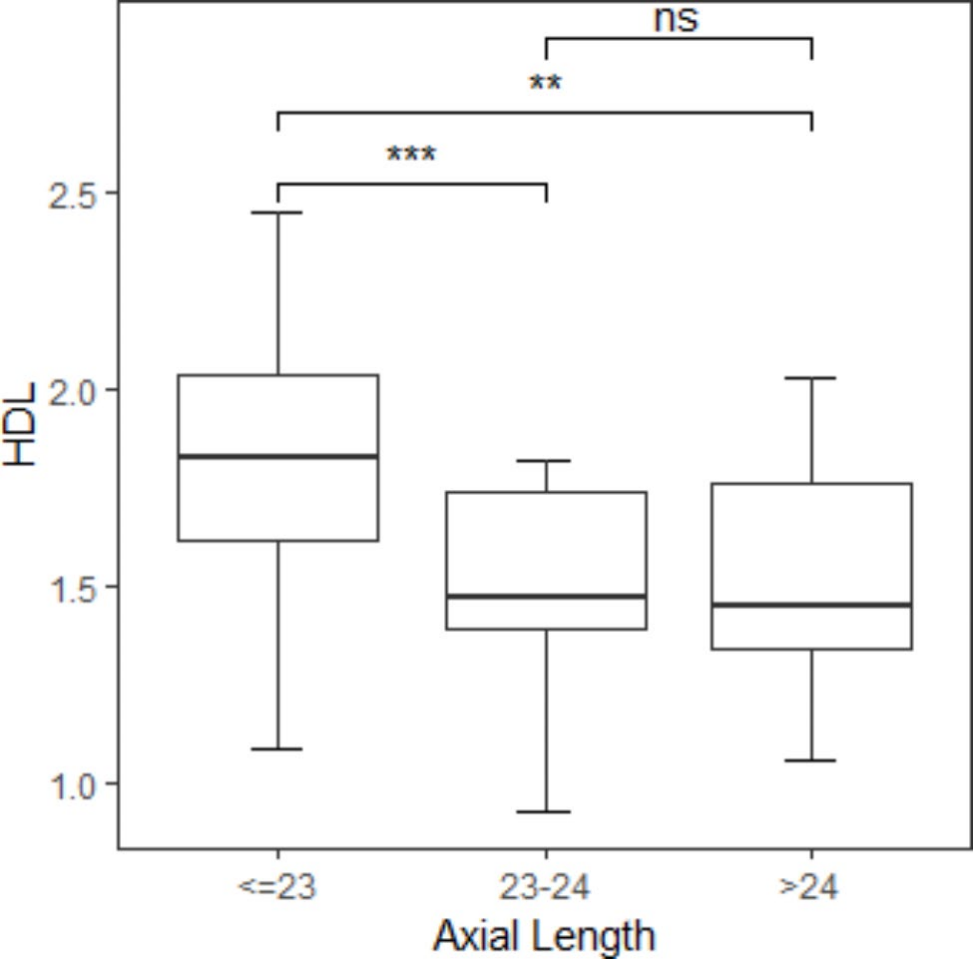
There existed significant differences between Group A (axial length < = 23 mm) and Group B (axial length 23-24 mm) (p = 0.00088). Significant differences were also found between Group A(axial length < = 23 mm) and Group C (axial length > 24 mm) (p = 0.0012). However, no significant differences were found between Group B (axial length 23-24 mm) and Group C (axial length > 24 mm) (p = 0.77)

**Fig. 2:**
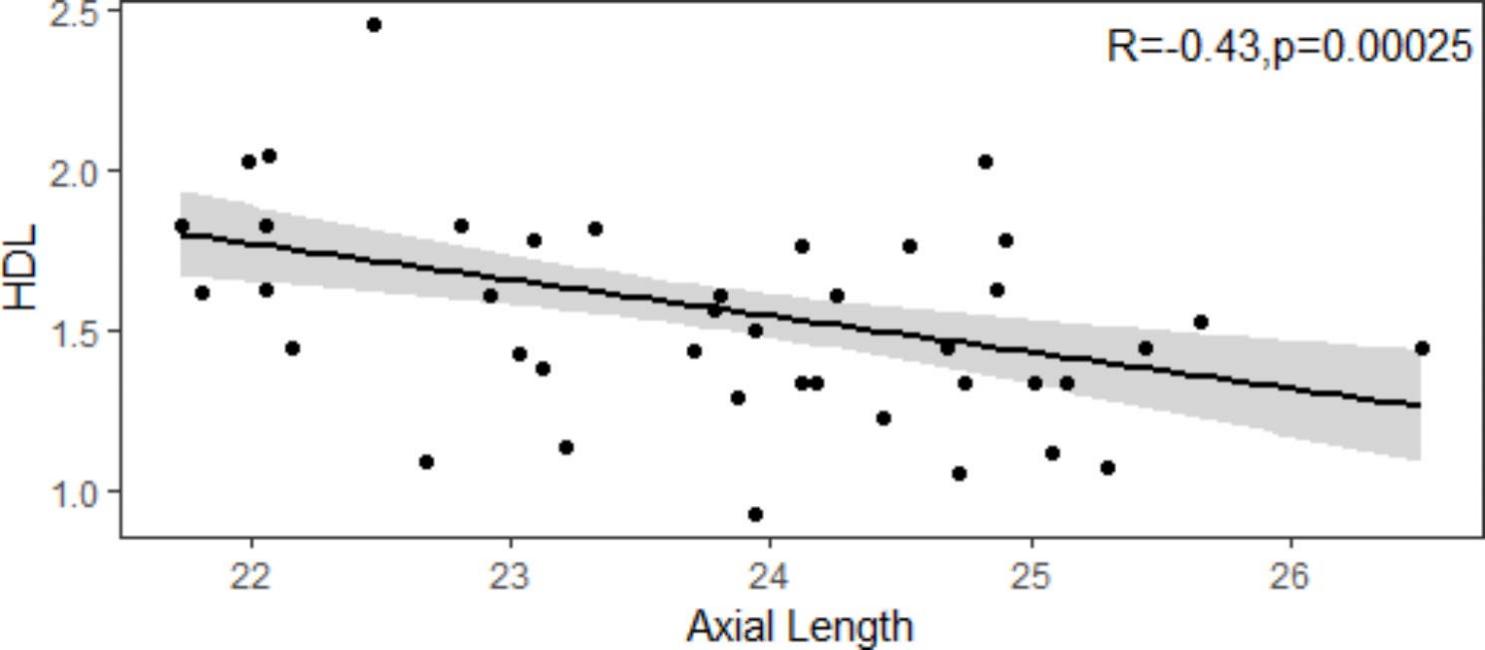
There was a statistically significant (p = 0.00025) and adverse (R = -0.43) association between axial length and HDL.

We found that there existed significant differences between Group A and Group B (*p* = 0.00088). Significant differences were also found between Group A and Group C (*p* = 0.0012). However, we did not find any significant differences between Group B and Group C (*p* = 0.77) (Fig. 1). Applying a Pearson Coefficient, we evaluated the association between axial length and HDL and discovered that there was a statistically significant (*p* = 0.00025) and adverse (R = -0.43) association between axial length and HDL (Fig. 2).

## Discussion

In our study, we found that there was a statistically significant difference in the HDL level between Group A (axial length < = 23 mm) and Group B (23 mm < axial length < = 24 mm). Group A had higher levels of HDL than Group B. It was also found that Group A had a considerably higher concentration of HDL than Group C (axial length > 24 mm). There was a statistically significant negative correlation between axial length and HDL levels.

Few studies have studied the association between axial length and HDL. The main protein component of HDL, apolipoprotein A-I (apoA-I), controls its size and structure, solubilizes its lipid components, removes cholesterol from peripheral cells, activates the LCAT enzyme, and transports the resultant cholesterol esters to the liver [23–25]. Most studies involve HDL put their attention on apoA-I due to its significance in the structure and function of HDL.

In 2006, Bertrand et al. revealed the “GO/GROW” and “STOP” signals in regulating eye growth [26]. The “GO/GROW” signal is believed to accelerate axial elongation, while the “STOP” signals is deemed to stop or prevent the growth of axial length. They demonstrated that apoA-I was capable of inhibiting excessive ocular axial growth, which enabled it to perform the function of a STOP signal. They have proved that apoA-I exhibits a STOP profile in both the retina and the fibrous sclera, and ocular axial elongation could be inhibited by up-regulating apoA-I. It is assumed that PPAR-α agonists increase the expression of apoA-I, the HDL lipoproteins’ main protein component, through transcriptional pathways [27, 28]. Numerous earlier investigations confirmed that PPARs positively regulated apoA-I. According to a research by Gervois et al., hepatic apoA-I and apoA-II expression was induced by PPAR-α activation, and this boosted plasma HDL cholesterol in humans [28]. It has also been demonstrated that PPAR-D can boost apoA-I and high-density lipoprotein production via activating the ATP-binding cassette transporter 1 (ABCA1) gene [29]. The findings of these investigations provided support for the hypothesis that apoA-I might be a common downstream target for the PPARs.

Plasminogen and Wnt pathways are two potential relevant signaling cascades that could be involved in regulating ocular axial development downstream of apoA-I [30, 31]. Through its inhibition of the plasminogen pathway, apoA-I may function as a STOP signal, deactivating matrix metalloproteinases and TGF-β and preventing their activation. There is a correlation between the attenuation of TGF-β and axial elongation [30]. Additionally, apoA-I has the potential to influence plasminogen activation via the low density lipoprotein receptor (LDLR) family [32]. On the other hand, Wnt signal transduction pathway plays a vital role in the development of the eye by the coreceptors [33, 34]. It is well established that MFRP (membrane Frizzled-related protein) is connected to the Wnt binding cysteine-rich domain of the frizzled family of transmembrane proteins [35]. These proteins are competitive inhibitors of Wnt signaling, which is an important mechanism in the formation of the vertebrate eye. These findings provide evidence that apoA-I plays a role in the transmission of the STOP signal, which supports the hypothesis that apoA-I is involved in this process.

There are some effective ways to raise the levels of HDL cholesterol. Getting more physical activity, losing extra weight, choosing healthier fat (monounsaturated and polyunsaturated fats), drinking moderate amounts of alcohol, stopping smoking, are demonstrated to be helpful in boosting HDL cholesterol.

This study was carried out within the scope of Hangzhou, a southern city in China, involving the analysis of children, which can be an epitome for the analysis of ocular status. As far as we know, no definite research was designed to study the association between HDL and axial length. Hence, the result in our study, negative correlation between axial length and HDL levels, may bring the HDL levels in the population of teenagers to the attention of ophthalmologists.

However, our study had some limitations. First and foremost, since our study is based on a cross sectional research, we can not determine whether there is a causal relationship between axial length and HDL or not. Also, the data in our study is gained from a single hospital, which may have a regional bias on the result. Furthermore, due to the fact that our study only included a limited number of kids, further large multicenter randomized controlled study is needed to assess the association and causal relation between axial length and HDL in children.

## Conclusions

In conclusion, we found that there was a significantly inverse relationship between axial length and the levels of HDL in children. Of course, further large multicenter RCTs will be needed to confirm our result.

## Data Availability

The data that support the findings of this study are available from the corresponding author (TS) upon reasonable request.

## Abbreviations

HDL: High-density lipoprotein
AL: Axial length
LDL: Low-density lipoprotein
SER: Spherical equivalent refraction
BCVA: Best-corrected visual acuity
logMAR: Logarithm of the minimal angle of resolution
